# Supplementary material for: QTLs and Candidate Genes for Seed Protein Content in Two Recombinant Inbred Line Populations of Soybean
Source: Plants (Basel). 2023 Oct 16;12(20):3589. doi: 10.3390/plants12203589 (PMC10610525; doi:10.3390/plants12203589)
Supplement: Supplementary file 1 [file plants-12-03589-s001.zip › Supplementary Tables_v3.pdf]

Table S1. Mean and range of the seed protein content (%) of the parental and recombinant inbred lines (RILs) in two mapping populations over three years.

Year	Parents			YS2035 × Saedanbaek (YS)			Saedanbaek × Ilmi (SI)		
				Protein content (%)			Protein content (%)		
	YS2035	SD	IM	RILs ± Sd	Range	H^2	RILs ± Sd	Range	H^2
2020	47.3	54.2	42.4	46.6 ± 3.37	37.4–55.3		45.1 ± 3.87	33.3–53.6	
2021	46.8	54.3	44.4	47.4 ± 2.87	40.8–55.4		48.0 ± 2.71	41.2–54.5	
2022	43.3	50.6	41.2	43.8 ± 3.37	36.3–53.6		45.7 ± 2.97	39.5–52.4	
Average	45.6	53.1	42.7	45.9 ± 2.78	39.4–52.3	0.84	46.5 ± 2.80	39.6–52.1	0.86

YS2035; YS2035-B-91-1-B-1, SD; Saedanbaek, IM; Ilmi, YS population; RIL derived from the cross between 'YS2035' and 'Saedanbaek'; n = 237; SI population; RIL derived from crossing between 'Saedanbaek' and 'Ilmi'; n = 189. Data represent the means ± Standard deviation (Sd). Significant differences between the trait values of the parental lines were determined using Student's t-test ($p < 0.001$). H^2; Heritability.

Table S2. Analysis of variance (ANOVA) for the soybean seed protein content of the mapping populations and their parental lines, 'Saedanbaek,' 'YS2035,' and 'Ilmi' in 2020, 2021, and 2022.

	YS2035 × Saedanbaek (YS)				Saedanbaek × Ilmi (SI)			
	Year	Genotype (RILs)	Year × Genotype	Residuals	Year	Genotype (RILs)	Year × Genotype	Residuals
DF[1]	2	236	460	1272	2	188	362	950
SS[2]	4761	15019	4852	283	2336	11458	2985	135
MS[3]	2380.5	63.6	10.5	0.2	1167.9	60.9	8.2	0.1
F value	10706.52	286.21	47.44		8242.9	430.2	58.2	
Pr (> *F*)[4]	<2 e-16	<2 e-16	<2 e-16		<2 e-16	<2 e-16	<2 e-16	
	***	***	***		***	***	***	

[1] Degrees of freedom. [2] Sum square. [3] Mean square. [4] Significant differences were determined at $p < 0.001$.

Table S3. Summary of the genetic linkage map of the RIL population derived from the cross between 'YS2035' and 'Saedanbaek'.

Ch[1]	LG[2]	Length (cM)	Total number of SNP marker	Number of Polymorphic markers	Number of SNP markers	Average distance (cM)
1	D1a	299	8935	1649	111	2.7
2	D1b	211	10,224	1137	109	1.9
3	N	330	8417	1606	124	2.7
4	C1	253	8638	1455	76	3.3
5	A1	300	8024	964	118	2.5
6	C2	313	9906	1047	115	2.7
7	M	233	8588	1361	90	2.6
8	A2	269	10,996	1256	110	2.4
9	K	253	8996	1859	132	1.9
10	O	302	9309	1344	107	2.8
11	B1	255	8412	1130	71	3.6
12	H	167	7708	721	87	1.9
13	F	215	10,885	1762	67	3.2
14	B2	241	7817	741	102	2.4
15	E	322	10,136	2019	166	1.9
16	J	245	7590	2252	206	1.2
17	D2	251	8906	1055	114	2.2
18	G	246	9957	1176	62	4.0
19	L	330	8719	2020	121	2.7
20	I	304	8212	1170	166	1.8
Average	-	267	9019	1386	113	2.5
Total	-	5339	180,375	27,724	2254	-

[1] Chromosome. [2] Linkage group.

Table S4. Summary of the genetic linkage map of the RIL population derived from the cross between 'Saedanbaek' and 'Ilmi'.

Ch[1]	LG[2]	Length (cM)	Total number of SNP marker	Number of Polymorphic markers	Number of SNP markers	Average distance (cM)
1	D1a	182	8935	2090	206	0.9
2	D1b	198	10,224	1302	219	0.9
3	N	172	8417	1308	194	0.9
4	C1	182	8638	1158	186	1.0
5	A1	161	8024	1542	235	0.7
6	C2	190	9906	1928	225	0.8
7	M	132	8588	963	120	1.1
8	A2	202	10,996	1288	201	1.0
9	K	143	8996	1646	175	0.8
10	O	162	9309	811	119	1.4
11	B1	218	8412	1256	197	1.1
12	H	140	7708	954	149	0.9
13	F	181	10,885	1726	197	0.9
14	B2	103	7817	793	128	0.8
15	E	147	10,136	1828	173	0.8
16	J	148	7590	1640	195	0.8
17	D2	141	8906	666	88	1.6
18	G	175	9957	1933	164	1.1
19	L	161	8719	2207	223	0.7
20	I	110	8212	857	150	0.7
Average	-	162	9019	1395	177	0.9
Total	-	3248	180,375	27,896	3544	-

[1] Chromosome. [2] Linkage group.

Table S6. QTLs associated with seed protein content identified in the recombinant inbred line populations derived from the cross between 'YS2035' and 'Saedanbaek' (Y×S) or 'Saedanbaek' and 'Ilmi' (S×I).

Population[1]	Marker[2]	Year	Chr[3]	Genetic Position (cM)	Marker Interval (Left-Right)	Physical Position of Markers (bp)[4]	LOD[5]	PVE[6] (%)	Add[7]
Y×S	***qPSD15-1***[8]	**2020**	**15**	**305**	**AX-90398867–**	**7,930,801–**	**14.0**	**17.5**	**-2.7**
		**2021**			**AX-90497794**	**8,678,412**	**14.6**	**17.1**	**-2.1**
		**Average**[9]					**12.3**	**13.8**	**-1.7**
	qPYS16	2022	16	245	AX-90317665–	37,189,961–	8.1	8.4	0.9
					AX-90406895	37,510,158			
	qPSD17	2020	17	157	AX-90313711–	13,052,551–	5.6	5.2	-0.8
					AX-90518221	13,148,914			
	qPSD18-1	**2022**	**18**	**75**	**AX-90492284–**	**46,911,930–**	**6.7**	**7.0**	**-0.9**
		**Average**			**AX-90474731**	**47,526,734**	**5.9**	**5.5**	**-0.6**
	qPSD18-2	2020	18	113	AX-90486233–	50,968,081–	7.6	7.2	-0.9
					AX-90413655	51,373,226			
	qPSD20-1	**2020**	**20**	**96**	**AX-90308709–**	**31,781,045–**	**21.1**	**22.5**	**-1.8**
		**2021**		**96**	**AX-90358193**	**31,961,695**	**24.7**	**29.1**	**-1.6**
		**2022**		**96**			**20.9**	**24.7**	**-1.8**
		**Average**		**96**			**30.6**	**35.4**	**-1.6**
S×I	*qPSD09*	2022	9	19	AX-90306520–	3,252,030–	6.2	3.8	0.6
		Average			AX-90353369	3,612,973	5.5	3.5	0.5
	qPSD15-2	2021	15	23	AX-90487528–	3,741,868–	7.5	5.4	0.6
					AX-90366042	3,757,358			

S×I	***qPSD20-2***	**2020**	**20**	**68**	**AX-90308709–**	**30,395,400–**	**23.0**	**34.1**	**2.4**
		**2021**		**68**	**AX-90445958**	**31,781,045**	**48.2**	**59.7**	**2.1**
		**2022**					**55.0**	**66.0**	**2.4**
		**Average**					**52.7**	**61.5**	**2.3**

[1] Y×S: YS2035 × Saedanbaek or S×I: Saedanbaek × Ilmi. [2] *qPSD*, 'Saedanbaek' contributed to the allele. [3] Chromosome (Chr). [4] Physical position of the marker interval. The soybean reference genome (*Glycine max* Wm82.a2.v1) was used to determine the physical positions of the markers. [5] Logarithm of odds value at the peak likelihood of QTL. [6] Phenotypic variation explained (PVE) by QTL. [7] Additive effect. [8] Markers highlighted by the bold font are main selected QTLs. [9] Average values for three years: 2020, 2021, and 2022.

Table S7. Characteristics of singe nucleotide polymorphisms (SNP) between 'YS2035', 'Saedanbaek', and 'Saedanbaek' and 'Ilmi' in the QTL regions for seed protein content identified on chromosome 15, 18, and 20.

SNP type	Ch15	Ch18	Ch20
	qPSD15-1	*qPSD18-1*	*qPSD20-1, qPSD20-2*
3'UTR variant	72	43	3
5'UTR premature start codon gain variant	7	4	2
5'UTR variant	42	25	11
Conservative inframe deletion	1	0	0
Downstream gene variant	112	73	2
Frameshift variant	9	6	1
Intron variant	178	85	2
Missense variant	96	91	55
Missense and splice region variant	0	0	2
Splice acceptor and intron variant	0	0	0
Splice donor and intron variant	4	0	0
Splice region and intron variant	8	0	9
Splice region variant	1	2	1
Splice region variant and intron variant	0	2	0
Splice region variant and synonymous variant	0	1	0
Splice region and synonymous variant	1	1	0
Start lost	0	0	0
Stop gained	4	3	1
Stop lost	0	0	0
Stop retained variant	1	0	0
Synonymous variant	73	35	2
Upstream gene variant	92	15	23
Total	701	386	114
